# The Governance of Unmanned Aircraft Systems (UAS): Aviation Law, Human Rights, and the Free Movement of Data in the EU

**DOI:** 10.1007/s11023-020-09541-8

**Published:** 2020-09-09

**Authors:** Ugo Pagallo, Eleonora Bassi

**Affiliations:** 1 Law School, University of Turin, Lungo Dora, Siena 100-10153, Turin, Italy; 2 Politecnico of Turin, Nexa Center for Internet & Society, Via Pier Carlo Boggio 65a-10138, Turin, Italy

**Keywords:** Aviation law, Co-regulation, Coordination mechanism, Data protection, Governance, Legal regulation, Middle-out approach, Soft law, Unmanned aircraft system (UAS)

## Abstract

The paper deals with the governance of Unmanned Aircraft Systems (UAS) in European law. Three different kinds of balance have been struck between multiple regulatory systems, in accordance with the sector of the governance of UAS which is taken into account. The first model regards the field of civil aviation law and its European Union (EU)’s regulation: the model looks like a traditional mix of top-down regulation and soft law. The second model concerns the EU general data protection law, the GDPR, which has set up a co-regulatory framework summed up with the principle of accountability also, but not only, in the field of drones. The third model of governance has been adopted by the EU through methods of legal experimentation and coordination mechanisms for UAS. The overall aim of the paper is to elucidate the ways in which such three models interact, insisting on differences and similarities with other technologies (e.g. self-driving cars), and further legal systems (e.g. the US).

## Introduction

Over the past 15 years, scholars have increasingly focused on the normative challenges of Unmanned Aircraft Vehicles (UAV), and Systems (UAS), also popularly known as drones. First, the attention was drawn to the military use of this technology since the mid 2000s, that is, during the first years of the second Gulf War in Iraq (and in Pakistan). Whilst, in his 2010 Report to the UN General Assembly, the Special Rapporteur on extrajudicial, summary or arbitrary executions, Christof Heyns, urged the then Secretary-General Ban Ki-moon to convene a group of experts in order to address “the fundamental question of whether lethal force should ever be permitted to be fully automated,” another UN Special Rapporteur, Philip Alston, declared that same year that “a missile fired from a drone is no different from any other commonly used weapon… The critical legal question is the same for each weapon: whether its specific use complies with IHL,” i.e. current international humanitarian law (in Pagallo 2013, at 4 and 59). A decade later, this kind of debate is still wide open.

The use of drones, however, can also affect the civil (as opposed to the state and the military) sector. This is the field under scrutiny in this paper. Scholars have examined matters of safety and security, drones market growth, public trust or distrust, up to the regulatory efforts of national and international lawmakers. Their latter aim is to reform current air traffic management systems, so that UAVs and UAS can gradually start sharing such air space with traditional aircrafts. In the US, for example, the 2012 FAA Modernization and Reform Act, i.e. Public Law 112-95, together with the FAA Reauthorization Act of 2018 provide for a federal legal framework, which is complemented by the powers of the Federal Aviation Administration (FAA) and, to some of extent, the States of the Union. In the European Union (EU), a similar path has been followed by Regulation 2018/1139 on common rules in the field of civil aviation. By repealing the previous Regulation 2008/216, the new set of rules reduces powers and competences of both Member States and national agencies on drones operations, by devolving most of the relevant ruling powers to the European Commission and to the European Aviation Safety Agency (EASA).

The aim of the paper is to restrict the focus of the analysis to the EU regulatory efforts in the civil sector, in order to cast light on different models of legal governance that EU lawmakers have adopted in the fields of civil aviation law, human rights protection, and data protection law with the free flow of such data. At the international law level, the notion of governance is usually related to “the formation and stewardship of the formal and informal rules that regulate the public realm, the arena in which state as well as economic and societal actors interact to make decisions” (Grindle 2007). Such formal and informal rules strike different forms of balance between multiple regulatory systems, such as the forces of the market and of social norms, between law, ethics, and technology. One of the main contentions of this paper is that such balances vary in accordance with the specific sector of the governance of UAS which is taken into account in EU law.

Next, Sect. 2 sets the level of abstraction of this paper, by distinguishing three forms of legal regulation with their variables. Then, Sect. 3 illustrates the centralized framework set up by Reg. (EU) 2018/1139 on civil aviation. In addition to the top-down approach of this regulation, special attention is drawn to the role that soft law plays in this context. Section 4 aims to widen our perspective, in order to comprehend the complex set of rights that are at stake with the civil use of UAS. In the phrasing of Art. 29 Data Protection Working Party’s 2015 Opinion on the use of drones, such rights regard “the indivisible, universal values of human dignity, freedom, equality and solidarity [and] the principles of democracy and the rule of law” (Art. 29 WP 2015). Section 5 deepens this analysis in light of a particular fundamental right, that is, personal data protection vis-à-vis its counterpart, i.e. the free movement of such data. The EU data protection regulation, or GDPR, offers an alternative model of legal governance, summed up with the principle of accountability, which can be conceived of as a sort of interface between traditional top-down approaches, as occurs in the field of civil aviation, and forms of self-regulation. Section 6 examines another model of governance adopted by the EU through methods of legal experimentation and coordination mechanisms in the field of UAS. Section 7 concludes the analysis on how such different models of governance may interact.

## On Legal Regulation and its Variables

Legal regulation is an essential ingredient of most notions and models of governance (Pagallo 2015). By taking into account the regulatory aims of the law, we should distinguish three kinds of legal regulation, i.e., between (i) traditional forms of top-down regulation, such as an act, or a statute, which mostly hinge on the threat of physical or pecuniary sanctions; (ii) manifold ways of self-regulation, or bottom-up approaches, with limited accountability and legal framing; and (iii) forms of co-regulation that can be understood as a sort of interface between top-down and bottom-up solutions, between legislators and stakeholders.

This basic demarcation between different forms of legal regulation and hence, of governance can be further developed with the variables of each observable of the analysis. As to the forms of top-down regulation, lawyers, especially in the international law field, distinguish between monistic and dualistic approaches. Monism refers to the functioning of a legal system—or to the interaction between two or more legal systems—which is ultimately based on a single legal source, such as the constitution of a state. Dualism has to do with the distribution of competences and coordination between two or more legal systems, each of which has its own constitution, or basic legal source. For example, it is still an open issue whether the EU law should be grasped either in monistic terms, or in a dualistic manner: the EU Court of Justice’s doctrine is monistic, whilst both the German and Italian constitutional courts endorse a dualistic approach. This alternative affects the international regulations of UAS as well. Such regulations comprise the Chicago Convention from 1944 with its Annexes (and subsequent amendments), much as the soft law provided by ICAO through its standards and recommended practices (Masutti and Tomasello 2018). Although the latter do not have the same binding force of the Convention, the Contracting States should collaborate in securing that their national regulations are uniform with such standards and recommended practices. This form of international cooperation, however, according to certain scholars, should be strengthened through the development of a proper international legal framework for UAS, due to the unique challenges brought forth by this technology and the need to develop and timely adopt new standards (Fiallos 2016).

Current debates on the international laws of UAS, regardless of the monistic or dualistic nature of this law, show nonetheless that such regulations leave room for different models of governance at the ‘regional level,’ e.g. the EU laws in the field of UAS. It is noteworthy that all the legal sectors under investigation in this paper, such as the fields of civil aviation law and of data protection, present a double level of top-down intervention, namely, that of the EU member states (national level), and that of the EU (international or quasi-federal level). Yet, it is up to the EU and its Member States to determine how this double level of top-down intervention should actually work. After all, the EU lawmakers have adopted two different regulatory models in the field of UAS over the past 12 years, i.e. the fragmented and dual approach of Reg. (EC) 2008/216 and the centralized legal framework of Regulation (EU) 2018/1139. One of the main aims of this paper will be to complement the analysis of such models of top-down regulation, whether national or international, whether monistic or dualistic, with further forms of legal governance endorsed by the EU legislators in the field of UAS.

As regards the second observable of the analysis, i.e. the notion of self-regulation, there are multiple bottom-up solutions. For instance, according to Chris Marsden’s “Beaufort scale” (Marsden 2011), eight different levels of self-regulation can be singled out, from ‘pure’ unenforced forms of self-regulation, such as in Second Life (scale 0), to ‘approved’ self-regulation, as in Hotline (scale 8). These forms of limited accountability and legal framing are not particularly relevant in the context of UAS regulation and its governance. Rather, such bottom-up solutions should be scrutinized in connection with further forms of co-regulation, as a sort of legal link between legislators and stakeholders.

Yet, also the notion of co-regulation has its own variables. This interface between top-down and bottom-up approaches includes forms of approved compulsory self-regulation (e.g. ICANN), and scrutinized self-regulation (NICAM), down to independent bodies with stakeholder fora, in which top-down directives of the government are co-regulated through taxation and/or compulsory levy (Marsden 2011). In addition to these forms of co-regulation, we should take into account the accountability principle of the EU data protection regulation, the ‘GDPR’ (see below in Sect. 5); much as the coordination mechanisms of legal experimentation (Sect. 6). This differentiation between multiple forms of co-regulation is critical, since it allows us to understand how the bar is set between the ends of the regulatory spectrum, that is, between strict top-down and pure bottom-up regulations.

In light of this threefold class of legal regulation, we can say that each regulatory solution strikes a different kind of balance between multiple regulatory systems in competition. As mentioned above in the introduction, one of our main contentions is that different kinds of balance have been struck in the field of UAS, in accordance with the sector that is scrutinized under EU law. The next section examines the current state-of-the-art in civil aviation law, in order to pinpoint what model of legal governance the EU legislators have opted for in this field.

## On Civil Aviation in EU and its Model of Legal Governance

UAS operations in Europe are currently disciplined by Regulation (EU) 2018/1139 on common rules in the field of civil aviation, the so-called “new basic regulation.” The new set of rules repealed the dual approach of the previous 2008 regulation, i.e. Reg. (EC) 2008/216. According to this latter legal framework, the EU lawmakers only provided rules for UAS with an operating mass over 150 kg and expressly excluded the regulation of certain types of drones, either due to their activity or their weight. This means that each EU Member State and their national aviation agencies had regulatory powers for all the other kinds of drones throughout a decade. This meant however the fragmentation of the system. A number of extremely detailed regulations by multiple national authorities raised the risk of hindering this vibrant field of technological innovation. The swirl of administrative acts by the Italian civil aviation authority, i.e. “ENAC,” illustrated this deadlock in the mid 2010s (Pagallo 2017a).

In order to guarantee certainty, harmonization and clarification of the rules on drones, the new 2018 EU regulation sets up a centralized, top-down framework, in which the main ruling powers are devolved to both the European Commission and the European Aviation Safety Agency (EASA). The new regulation is adopted in the name of the subsidiarity principle. The latter governs the exercise of the EU’s competences, as laid down in the Treaty of the European Union (Article 5(3)), and applies to all the cases in which the Union has no exclusive competence, as for civil aviation. In the wording of the new Act, “since the objectives of this Regulation, namely establishing and maintaining a high uniform level of civil aviation safety, while ensuring a high uniform level of environmental protection, cannot be sufficiently achieved by the Member States because of the largely transnational nature of aviation and its complexity, but can rather, by reason of their Union-wide scope, be better achieved at Union level, the Union may adopt measures, in accordance with the principle of subsidiarity” (Rec. 88).

The new basic regulation concerns all drones regardless of their size and weight, although there are some exceptions, which are up to EASA to regulate with its guidelines, pursuant to Annex I and Art. 141(4) of the regulation. Member States can lay down specific national rules for UAS, either by granting specific exemptions to some European requirements, or amending the implementing and delegated acts of the Commission, in accordance with Art. 56(8) and 71 of the regulation (Bassi 2019a). However, the aim to guarantee standards for the safety, efficiency and environmental impact of air traffic—so that drones can gradually begin to share the air space—is mostly defined at the EU level. Similarly to the US regulatory model, which mostly revolves around the powers of the Congress and the Federal Aviation Administration, the regulatory powers of the EU are devolved both to the Commission and to EASA. Since 2019 onwards, scholars had thus to pay attention to the European Commission’s implementing and delegated acts, mandated by Reg. 2018/1139. Such acts establish a specific set of detailed rules for different classes of UAS operations (Bassi 2020). Examples are the Delegated Regulation (EU) 2019/945 on unmanned aircraft systems and on third-country operators of UAS, and the Implementing Regulation (EU) 2019/947 on the rules and procedures for the operation of unmanned aircrafts, as amended by the Commission Implementing Regulation (EU) 2020/639 of 12 May 2020, related to standard scenarios for operations executed in or beyond the visual line of sight.

In addition, there are the regulatory powers of EASA. They are both hard and soft. As to the hard tools of EASA, pursuant to Art. 75(2)(b) of Reg. 1139/2018, the Agency has the power to develop, upon request of the Commission, technical rules that cannot be changed by the Commission without prior coordination with the Agency. This power of EASA is disciplined by Article 115(1) of Regulation (EU) 2018/1139 and by an ad hoc internal ‘Rulemaking Procedure’ (EASA 2015) (MB Dec. No 18-2015). As regards the soft powers of EASA, the Agency can issue such acts, as the Guidance Material and Acceptable Means of Compliance that flesh out the measures to comply with the regulation, including e.g. the description of the methodology for conducting a Specific Operation Risk Assessment and the model of a pre-defined risk assessment.

Two basic features of this regulatory model of governance for UAS in the civil aviation field can be further stressed in light of the current regulation of autonomous ground vehicles (AVs), or self-driving cars. Although UAS and AVs may look somehow similar, the ways in which they are disciplined in the EU suggests some striking differences. In addition to technological and geo-political reasons (Pagallo 2011), such different regulatory approaches concern alternative models of top-down regulation, and their interplay with the soft tools of the law. As to the different types of top-down regulation, in addition to Regulation (EU) 2018/113 in the field of civil aviation, there is a set of common rules established at the EU level also in the field of AVs. The list includes both a regulation on the approval and market surveillance of motor vehicles, and three directives on liability for defective products, the sale of consumer goods, and insurance against civil liability (Pagallo et al. 2019). Contrary to the field of UAS, however, the most critical legal issues of current traffic law depend on the legislation of each EU member state, as occurs with matters of redress, damages, or tortuous liability. We are far from even beginning to imagine a quasi-federal legal framework for the use of AVs at the EU level. All the amendments which have been made to existing traffic laws, in order to allow for the testing and use of driverless technology on public roadways, are up to national legislators: Spain passed its own law with the Dirección General de Tráfico from November 2015; Belgium with the Royal Order from March 2016; Italy with the “Smart Road” decree from February 2018; France with the norms on “la croissance et la transformation des entreprises” from April 2019; and so on. Although both regulatory models of civil aviation and road traffic laws are thus top-down and dualistic—because there is a distribution of competences between the EU and its member states—only the regulatory framework of UAS appears highly centralized.

A second crucial difference between UAS and AVs, and hence, another crucial facet of the EU regulatory model of governance for UAS has to do with the role of soft law. The lack of any robust soft law for AVs, as a matter of fact, appears as the by-product of an on-going process to determine the rules of hard law in that field. As regards the governance of UAS, the soft powers of EASA can hardly be overestimated. They are established by Articles 75 and 76 of the basic regulation, and comprise (i) opinions and recommendations on the current legal framework; (ii) the development of standards for the integration of UAS operations in the single European sky strategy; (iii) monitoring functions that regard the application of the 2018 regulation; and, (iv) the coordination of the activities by member states, which includes certifications, duties of oversight—in particular cooperative and cross-border oversight—and enforcement tasks (Bassi 2020).

Some of these soft powers of EASA on e.g. development of standards can be properly conceived of as the middle ground between the top-down regulatory approach illustrated thus far, and the forces of the market. According to a study of the EU institutions, the drone services market is going to grow noticeably, with estimates “between €10bn by 2035 and €127bn for the coming years” (European Commission 2017). Yet, such growth would be impossible without efforts of coordination and cooperation with the drone industry. Going back to EASA’s Guidance Materials and Acceptable Means of Compliance, it is remarkable that the principal aim of such acts is to assist operators, for example, when applying for an authorization in the specific category of the operation to be performed. In the description of the rulemaking procedure followed for the adoption of its Opinion 5/2019, EASA has stressed that the definition of standard scenarios for specific drones operations is developed on the basis of the “in-service experience of some Member States.” Stakeholders and national experts of different member states are involved in the process (EASA 2019).

The EU top-down regulatory approach to the field of civil aviation is thus crucially complemented, all in all, by the soft tools of the law. Soft law represents the interface between the common standards on safety, efficiency and environmental impact of the air traffic—as the main goals of the current reform of the air traffic management system in Europe—and the role that the forces of the market play in this context. The overall aim of the EU lawmakers is to attain that the whole framework, including UAS sharing the air space with traditional aircrafts, is at full speed by 12 September 2023, i.e. as established in Article 140 of the 2018 Regulation.

Still, the governance of UAS and the legal regulations of the sector regard also but not only the field of civil aviation. UAS affect further fields as different as public security legislation, telecommunication and data protection law, product liability, criminal law, or insurance law (Custers 2016). Attention should be drawn as well to the impact of UAS operations on the protection of people’s rights, such as the right to dignity and freedom of assembly and association, privacy and non-discrimination, down to the criminal safeguards of the individuals (Finn and Wright 2012). The next section examines what model of governance may follow as a result of this broader view on the normative impact of UAS.

## When Drones Meet People’s Rights

Scholars and authorities—such as the Art. 29 Working Party, mentioned above in the introduction—have time and again stressed threats and challenges triggered by the use of drones. Such threats include a “chilling effect; dehumanisation of the surveilled; transparency and visibility, accountability and voyeurism; function creep; bodily privacy; privacy of location and space; and privacy of association” (Finn and Donovan 2016).

The provisions and legal safeguards that are hence at stake with the use of drones regard acts and statutes of national states with their constitutions, much as international conventions and agreements. In Europe, for example, attention should be drawn to a long-standing tradition, which is defined by the 1950 Convention on Human Rights (“ECHR”), and the 2000 EU Charter of Fundamental Rights (“CFR”). In the case of the ECHR, the legal reference is to the human nature of such rights, in accordance with the terminology of international lawyers and due to the international nature of the convention. In the case of the CFR, the reference is to the fundamental character of the rights, because of the constitutional relevance of the Charter in the system of legal sources in the EU. On this basis, scholars have examined whether this set of rights, both “human” and “fundamental,” can properly tackle the normative challenges brought about by the use of drones in the civil sector, or whether further advancements in the technology, e.g. the use of highly sophisticated AI drones, may fall within the loopholes of the legal system, as occurs, for example, in the field of the laws of war and of international humanitarian law (Pagallo 2013).

Such alternative on either opting for the enforcement or the amendment of today’s drone regulations in the civil sector does not seem to affect, however, the model of legal governance illustrated so far. On the one hand, the enforcement of today’s laws by national and international courts, such as the European Court of Human Rights in Strasbourg, or the EU Court of Justice in Luxembourg, complements the top-down rules set up by governments and legislators through the case law of such courts. This is the approach of the Art. 29 Working Party in the 2015 Opinion on the use of drones (wp231), as mentioned above in the introduction. In that Opinion, the EU data protection authorities insisted on how UAS operations should abide by the “universal values of human dignity” (Protocol 13 to the ECHR and Art. 1 of the CFR); “freedom” (Section I of both the ECHR and the CFR); “equality” (ECHR’s Protocol 12 and CFR’s Art. 20); and so forth.

On the other hand, we may admit that current advancements in technology will require a new generation of rights and principles, in addition to the list enshrined in national constitutions and international agreements. For example, by taking into account the normative challenges that are unique to AI, scholars have stressed the limits of traditional principles, such as justice and autonomy, beneficence and non-maleficence, and hence, the need of enabling such principles through a new one: the principle of “explicability” (Floridi et al. 2018). Even in this case, we should concede however that the top-down regulatory model discussed in the previous section would not be challenged. Whilst current discussions on the ethical and legal principles of AI and of other emerging technologies revolve around whether and to what extent policy makers and legislators have to endorse a new set of principles and rights, the ultimate end is to make both new and old rights enforceable. Therefore, should our conclusion be that the protection of people’s rights, vis-à-vis the use of drones in the civil sector, does not entail any new form of legal governance?

We think there is a relevant ‘exception.’ It regards the field of data governance and the corresponding right to personal data protection in EU law. Article 132 of the civil aviation regulation includes a safeguard clause for privacy concerns, which refers to the application of the General Data Protection Regulation (GDPR) Reg. (EU) 2016/679 and of the Regulation (EC) no. 45/2001 (repealed by Reg. (EU) 2018/1725). This does not mean that every UAS operation necessarily entails the processing of personal data, yet, manifold UAS applications for public or private surveillance, disaster relief or medical assistance, journalism or simple leisure, up to a fascinating variety of commercial services do involve the collection and processing of personal data (Art. 29 WP 2015). Accordingly, scholars have examined the several ways in which drone operators and manufacturers should comply with both constraints and principles of the GDPR, such as the purpose limitation principle, data minimisation, individual consent, storage limitation, and so forth. The attention has been also drawn to the data protection impact assessments set up by Art. 35 of the GDPR, and how the latter may relate to the operational risk assessment of Art. 11 Reg. (EU) 2019/947 on rules and procedures for the operation of unmanned aircraft (Bassi 2020). On top of that, a growing amount of work has been devoted to the implementation of Art. 25 of the GDPR, namely, how to set up a new generation of GDPR-abiding drones in accordance with both the principles of data protection by design, and by default (Bassi et al. 2019).

Notwithstanding this amount of work on UAS and data protection, there are still few studies on the model of legal governance set up by the GDPR (Pagallo et al. 2019); and moreover, on how this model may relate to that which was under scrutiny above in the previous section, i.e. the model of legal governance for UAS in civil aviation law. The next section aims to fill this gap in today’s research.

## On Personal Data Protection and its Governance in EU Law

The GDPR is a long and complex legal text, which includes 173 recitals and 99 articles, some of which appear rather vague or opaque. According to certain scholars, “the GDPR can be a toothless or a powerful mechanism to protect data subjects dependent upon its eventual legal interpretation: the wording of the regulation allows either to be true” (Mittelstadt et al. 2016; Pagallo 2017b).

The overall architecture of this regulation looks however clear. The model adopted by the EU legislators, pursuant to the definitions illustrated above in Sect. 2, is a co-regulatory model of legal governance. The legal link between the top-down norms of the regulation and the self-regulatory choices of data controllers is given by the accountability principle enshrined in Art. 5 of the GDPR. On the one hand, Art. 5(1) lists six sets of principles that should be implemented by data controllers. These principles regard (i) lawfulness, fairness, and transparency of data processing; (ii) purpose limitation; (iii) data minimization; (iv) accuracy; (v) storage limitation; and, (vi) integrity and confidentiality. On the other hand, Art. 5(2) leaves room for self-regulatory measures, both technical and organizational, on the part of the data controllers, as to how they should attain the outcomes established by Art. 5(1), under the supervision of public guardians. Although not mentioned, the principle of accountability is similarly at work with the provision of Article 24(1): “the controller shall implement appropriate technical and organisational measures to ensure and to be able to demonstrate that processing is performed in accordance with this Regulation.”

The overall idea of the GDPR’s co-regulatory model is that personal data processing is a risky activity and nobody, better than data controllers know how to properly tackle the risks of their own data processing. This is why the logic of the accountability principle also operates with the provisions of Art. 25(1) on the principle of data protection by design and by default, of Art. 32 on the “security of processing,” and of Art. 35 on data protection impact assessments. Safeguards should indeed be pre-emptive, rather than simply remedial, and do not regard just those data subjects concerned by the processing, much as design solutions and organizational measures should abide by all the requisites of the regulation (Pagallo et al. 2019).

We may thus wonder how the co-regulatory model of data governance set up by the GDPR relates to the top-down regulatory approach of the EU civil aviation regulation in the field of UAS. A sound hypothesis suggests that we should grasp both regulations EU—2016/679 (i.e. data protection) and 2018/1139 (i.e. civil aviation) as complementary. This means that UAS operators, on the one hand, shall always abide by the top-down rules on e.g. safety and security established by the EU legislators in the field of civil aviation—eventually with the assistance and guide of EASA, as regards certifications and means of compliance—whereas, on the other hand, if such UAS operations entail the processing of personal data, it is up to the data controller to organize itself, in order to comply with the six sets of principles enshrined in Art. 5(1) of the GDPR.

The greater flexibility of the co-regulatory approach of the GDPR—vis-à-vis the top-down regulations of EU-2018/1139—may depend on two main facts. First, the speed of innovation that mostly distinguishes the field of data-driven technologies has suggested more adaptability than the top-down and soft law approach to civil aviation. Second, the long and well-established tradition of safety design assurance in the field of civil aviation has supported the most rigid approach of the corresponding regulation. However, in both fields of data protection and civil aviation, legislators have to address the common problem to design a set of rules, which should neither hinder the advance of technology, nor require over-frequent revision to tackle such a progress (Pagallo 2017c). We return to this problem of techno-regulation below in the next section.

The complementarity hypothesis on how to grasp the interaction between regulations EU—2016/679 (i.e. data protection) and 2018/1139 (i.e. civil aviation), has still some problems. The first open issue brings us back to the data protection impact assessments set up by Art. 35 of the GDPR and the operational risk assessment of Art. 11 Reg. 2019/947. Here, we face a chicken and egg dilemma. According to the GDPR, an impact assessment (“DPIA”) is mandatory when personal data processing entails high risks for the rights and freedoms of natural persons, “in particular using new technologies” (Art. 35(1)). Likewise, Art. 18(h) and (i) of Reg. 2019/947 impose on each member state a twofold duty, namely, to develop a “risk-based oversight system” for certain UAS operators and an “audit planning based on the risk profile, compliance level and the safety performance of UAS operators.” As a result, should the latter audit presuppose a UAS DPIA pursuant to Article 35 of the GDPR, or the other way around?

The second problem of the complementarity hypothesis regards the limits of the GDPR. As stressed above in Sect. 3, UAS operations do not only concern the processing of personal data, but also public security legislation and criminal law, rules on product liability and insurance law, telecommunication regulations, down to the processing of non personal data. Some of these issues shall be regulated under the “U-space services” developed by EASA, as the latter stressed in its draft for a Commission Implementing Regulation on a high-level regulatory framework (EASA 2020b), proposed in Opinion 01/2020 (EASA 2020a). Still, how the norms on civil aviation should relate to the complexity of such other fields of the current legal regulatory framework remains often unclear (Bassi 2020). Some of these fields, e.g. tortious liability, mostly fall within the regulatory powers of EU member states, so that risks of fragmentation are high.

A third trouble with the complementarity hypothesis concerns how the top-down approach of the civil aviation regulation can cope with the advancement of technology vis-à-vis the more flexible co-regulatory approach of the GDPR. Section 3 has already mentioned the role of EASA’s soft law in developing rules and standards for the integration of UAS operations within the Single European Sky strategy, and yet, this approach does not seem sufficiently adaptable. Even EU legislators and policy makers increasingly admit this (Pagallo et al. 2019).

Is there any further model of governance that can help us tackle the intricacies of technological innovation?

## Legal Experimentations and Data Governance

The aim of technological regulation should be to strike a fair balance between the protection of people’s rights and interests, on the one hand, and the development of sound technological research and innovation, on the other. Over the past years, scholars—and even more importantly, legislators and policy makers—have increasingly noticed that the more technology is complex, the less traditional top-down approaches are fruitful, in order to properly address the normative challenges of technology. Scholars and policy makers have accordingly examined alternative ways to govern the manifold fields of technological innovation.

The previous section has examined one of such alternative ways, i.e. the co-regulatory model of data governance set up by the GDPR with the principle of accountability. A problem with this approach, however, regards its limits. We often lack a set of common principles to be enforced in many vibrant fields of technological research, as occurs with the six sets of principles enshrined in Art. 5(1) of the GDPR. The on-going debate on the ethical principles of Artificial Intelligence (AI) and the consequent amendments to the current legal framework suggest that we can hardly transplant the co-regulatory model of the GDPR into other domains of technological regulation. Scholars and policy makers have thus considered further forms of regulation between top-down and bottom-up solutions. Section 2 mentioned some of them, such as forms of approved compulsory self-regulation, scrutinized self-regulation, or the setting of independent bodies with stakeholder fora. Still, these co-regulatory forms of legal governance fall often short in coping with crucial features of today’s technology, such as the lack of data about the probability of events, consequences, and costs, which should allow us to determine the level of risk (Pagallo 2017a). EASA has denounced this lack of data in its Opinion 01/2020.

The Agency proposed a “High-level regulatory framework for the U-space,” which includes the impact assessment presented by EASA for the draft of the proposed regulation. As the Agency admits, “as there is no sufficient data to perform a through quantitative safety risk assessment of the proposed regulation, EASA will use a general qualitative approach to conduct the safety risk assessment of the options analysed in this impact assessment.” According to the Agency, we should add to this lack of data the lack of a “common data exchange infrastructure.” In the Opinion of EASA, the idea is that such constraints should be addressed through monitoring procedures. The regulatory model of governance we are looking for should indeed provide the legal basis for collecting the empirical data and knowledge necessary for making rational decisions on a number of critical issues, for example, in order to better appreciate the threats associated with a certain technological application, such as a urban drone flight, a bunch of self-driving cars, or a team of service robots. This kind of factual information is a necessary condition for every sound model of legal governance today (Pagallo 2017a). In the wording of EASA, it’s crucial “a continuous and systematic process of data collection and analysis about the implementation/application of a rule/activity. It generates factual information for future possible evaluations and impact assessments. It also helps to identify actual implementation problems and support regular updates of the regulatory framework” (EASA 2020a).

Another crucial ingredient of the model has to do with the role of stakeholders and how they should be involved: we already stressed this point above in Sect. 3, in connection with EASA’s soft law tools and the development of standards in the field of UAS. The increasing use of drones for journalism and surveillance, medical assistance and commercial services, or just for fun and leisure, suggests that we should take into account the role of social standards, in addition to the development of technological standards. As shown by the use of drones during the Covid-19 crisis in urban areas, UAS clearly affect how people perceive and live in public and even private spaces. Consultations with stakeholders and forms of participation related to the use of drones can increase both the awareness of social benefits and knowledge of best practices and recommended behaviours for diminishing risks for safety and privacy. A sound model of governance for this field should thus include forms of involvement for the alignment of societal values and comprehension of public opinion, much as responsible experimentation could improve our understanding of how highly sophisticated technological systems may satisfy human needs (Bassi 2019b).

Remarkably, this latter approach to experimentation has been progressively adopted by policy makers and governments over the past two decades (Pagallo 2017a). The Japanese government, for instance, has created a number of special zones for the empirical testing and development of robotics and AI systems. Such forms of living labs, or *Tokku*, have concerned so far the fields of road traffic laws (at Fukuoka in 2003), radio law (Kansai 2005), data protection (Kyoto 2008), safety governance and tax regulation (Tsukuba 2011), much as road traffic laws in highways (Sagami 2013). These forms of legal experimentation have been developed in Europe, much as in US as well. Experiments have been particularly popular in the field of self-driving cars, although it is unsurprising that this trend has rapidly extended to the drone sector as well. In January 2019, the first special zone for drones in open labs was established in the harbour of Antwerp, so as to test the development of interoperability standards for communication systems. Further open labs have been created in other European cities, such as Turin, where an Open Lab is devoted—also but not only—to clarify the content of the rules and standards that privacy-friendly civilian UAS operations should abide by (Bassi 2020).

Legal experiments can be perfomed through forms of experimentation by derogation, by devolution, or by “open access,” that is, “allowing alternative lawful (collaborative) self-regulatory practices to arise” (Du and Heldeberg 2019). What all these kinds of legal experiments have in common regards their mechanisms of coordination (Pagallo et al. 2019). As a model of smart governance, such coordination mechanisms represent the interface between the top-down regulatory efforts of legislators and the bottom-up solutions of self-regulation. In particular, going back to the co-regulatory models introduced above in Sect. 2, the coordination mechanisms of legal experimentation set the regulatory bar between the accountability principle of the GDPR and every model of self-regulation, either as a form of approved compulsory or scrutinized bottom-up approach. The bar is lower than the accountability principle’s, because the coordination mechanisms of legal experimentation lack a set of common values, such as the six sets of principles enshrined in Art. 5(1) of the GDPR. The regulatory bar is higher than in every model of self-regulation, because the perimeters of legal experimentation are defined by the legislator in a top-down way, that is, through e.g. public authorizations for security reasons, formal consent for the processing and use of personal data, mechanisms of distributing risks via insurance models and authentication systems, and more (Pagallo 2017c).

We can thus wonder how this governance model of coordination in the field of UAS may relate to the previous models illustrated so far, i.e. the top-down approach of civil aviation law supplemented by the protection of people’s rights (as seen above in Sects. 3 and 4), together with the co-regulatory approach of the GDPR (illustrated in Sect. 5).

A well-established tradition in computer science suggests a solution through the middle-out approach (Pagallo et al. 2019). Both computer sciences and practical sciences such as the law have to address the constraints that arise during the design process when upgrading existing systems. This is the case of the middle-out design for human–computer interaction in urban spaces (Fredericks et al. 2016), or when building reference ontologies for the legal domain (El Ghosh et al. 2016). The same holds true in the field of UAS. The upgrading of the system through experiments and methods of coordination shall go hand-in-hand with the normative constraints set up by both the regulations on civil aviation and data protection. Such experiments are in fact conducted in legally de-regulated special zones through the set of coordination mechanisms that define the interface of the governance model. Interestingly, this approach to what is also dubbed as “experimentalist governance” (Zeitlin 2015), is at work with further initiatives in the field of data governance. Consider the European Commission’s policy on better and smart regulation (European Commission 2015), and the EU Better Regulation scheme for interoperability (TOGAF 2017), in which the use of participation schemes and coordination mechanisms can be understood as the interface of the model between top-down and bottom-up solutions. In addition, the approach is consistent with the stance on the rule of law taken by standardisation agencies and some governance models in the business field (Pagallo et al. 2019; Poblet et al. 2019).

This convergence is unsurprising. The field of UAS and its governance have shown that the more technological regulation is complex, the less top-down and bottom-up approaches are fruitful, and the more we should pay attention to forms of co-regulation through the middle-out level of the analysis. The time is ripe for the conclusions of our study.

## Conclusions

The paper has examined three models of governance for UAS in EU law for the civil sector, namely:

(i) The top-down model of civil aviation law, supplemented by both the tools of soft law and the legal safeguards for the protection of human and fundamental rights. This model highlighted both convergences between legal systems (e.g. the EU and US general laws on civil aviation), and differences between technologies (e.g. the decentralized regulation of self-driving cars in the EU vis-à-vis the centralized EU governance of UAS);
(ii) The co-regulatory model of data protection with the accountability principle enshrined in Art. 5 of the GDPR, which applies to all processing of personal data in the EU, regardless of the technology under scrutiny; and,
(iii) The middle-out model of coordination mechanisms for legal experimentation, which has been increasingly adopted by most legal systems to tackle the challenges of technological innovation.

The three models can be grasped according to a sort of legal spectrum. At one end of the spectrum, there are the strict top-down regulatory approaches that aim to govern both social and individual behaviour through the threat of physical or pecuniary sanctions, whereas, at the other end of the spectrum, we find pure self-regulatory solutions with limited accountability and legal framing. In light of the three models of UAS governance in civil aviation, data protection, and legal experimentation, we can thus say that the bar of legal regulation is progressively lowered as we move from the first to the second model, i.e. from civil aviation to data protection; and from the second to the third, i.e. from data protection to legal experimentation. The reason why the regulatory bar is progressively lowered depends on the flexibility that is necessary to properly deal with the normative challenges of UAS technologies.

By lowering the regulatory bar, from strict top-down solutions (e.g. aircraft security), towards more flexible co-regulatory approaches (e.g. personal data processing by UAS), it does not follow that the bar of legal safeguards is lowered as well. In the case of the GDPR’s model of data governance, the sets of principles of Art. 5(1) flesh out the outcomes that data controllers should attain under the supervision of public guardians. As regards the coordination mechanisms of legal experimentation, lawmakers determine the boundaries of the legally de-regulated special zones. As previously stressed in Sects. 5 and 6, the governance models for UAS operations should be grasped as complementary and in accordance with the goal which is taken into account, e.g. safety, efficiency, or environmental-friendly impact of UAS operations (first model); fair processing of personal data (second model); or empirical testing for new standards (third model).

The complementarity hypothesis leaves some issues open. Three of them are particularly relevant for the governance of UAS. First, each model of governance is still in progress. The civil aviation legal framework should be completed within 2023; data protection rules are often open to different interpretations; whereas legal experimentalism is instrumental to find out solutions for the previous models. Second, we mentioned that it is still unclear how such regulatory models should complement each other under certain circumstances, e.g. the impact assessments set up by Art. 35 of the GDPR vis-à-vis Art. 11 of Reg. 2019/947 on civil aviation. Third, further regulatory issues are not covered by such models. These gaps concern either the EU law and its interaction with the legal systems of the member states, or between these latter legal systems with problems of coordination. Gaps include also but not only public security legislation and criminal law, rules on tortious liability and some aspects of insurance law. Problems of fragmentation follow as a result of the distribution, or coordination of regulatory powers, up to 27 different member states.

This threefold set of open issues reminds us of the troubles of the law when dealing with the complexity of technology. The intricacy is corroborated by the threefold approach endorsed so far by the EU institutions for the governance of UAS. The complementarity and flexibility of the interaction between models may represent the only way in which the law can strike a fair balance between the protection of people’s rights and the development of sound technological research. In light of such a balance between safety and security, data protection and standards, environmental-friendly impact of UAS operations and the protection of human and fundamental rights, we should conclude that the governance of UAS is such a complex field of legal regulation that needs no single model, but three.
